# Introduction to The Special Issue “Safety Training Effectiveness: A Research Agenda”

**DOI:** 10.3390/ejihpe12100106

**Published:** 2022-10-19

**Authors:** Federico Ricci, Fabrizio Bracco

**Affiliations:** 1 Department of Biomedical, Metabolic and Neural Sciences, University of Modena and Reggio Emilia, 41121 Modena, Italy; 2 Department of Education Sciences, University of Genova, 16126 Genoa, Italy

This Special Issue, “Safety Training Effectiveness: A Research Agenda,” aims to address training as one of the many elements that play a role in determining so-called safety outcomes. The goal is to provide researchers, corporate decision-makers, government agencies, and international bodies with tools to increase hazard prevention in the workplace. This is fundamental given the unacceptable number of accidents that still occur every day across the world.

To this end, we define safety as the presence of conditions that make favorable outcomes likely, rather than the absence of unfavorable outcomes, as we turn our gaze toward Safety-II. A Safety-II perspective has no room for simplistic visions, for example, those that see human factors as the first or only cause of accidents and injuries. The causes of unfavorable outcomes can vary widely. Indeed, human beings are not the problem but the solution, for they can create the conditions that make favorable outcomes more likely and guarantee the improved functioning of complex socio-technical systems.

We do not wish to focus on whether training is an effective means of improving human performance; to do so would require us to consider a single component of the system (a Safety-I scenario)—the human being—rather than the system itself. We argue that effective safety training does not mean overcoming the weakness of the human component of systems. Our position is not rooted in blame, nor does it refer to simplistic models of the determinants of human performance.

We often witness, at least in production contexts, a form of Safety-I disguised as Safety-II, where training offers an alternative to the blame approach. After an adverse event, safety managers might naively consider an advanced, no-blame strategy (rather than simply punishing operators) as sufficient; they might propose training interventions that reiterate the importance of procedures and show workers how to correct their recklessness. Unfortunately, this is a surreptitious form of Safety-I (i.e., reactive, rather than proactive) because it targets people, not systems. It is also counterproductive because it delegitimizes training (a beneficial form of intervention when it is well-designed and planned). When workers perceive a training event to be a pointless and paternalistic review of procedures, they develop a distrustful and resistant attitude to training in general, even when it is well-designed.

We believe that researchers should shed light on the kind of training that contributes to producing safer systems through safety tuning, as Erik Hollnagel has proposed [1]. Safety tuning refers to the multiple functions that have to be finely tuned to ensure expected and acceptable performance. Systems cannot be made safer if they do not successfully manage the conditions in which people work. We are interested in explaining how training can contribute to this fundamental objective. A suitable metaphor might be that of a SIM card (i.e., training) that only works when it is inserted into a device (i.e., the organization) for which it is designed, and vice versa. Training must be tailored to the organization if either is to function optimally.

To this end, Mariani et al. [2] discuss the relationship between the effectiveness of training methods and the degree of trainee involvement, a subject highlighted in the most recent systematic reviews and meta-analyses. The authors present the validation of a newly developed measure of individual safety training engagement (STE), which comprises five elements relating to the dedication to and absorption of safety training in the trainee.

Sarpy and Burke’s article is closely related to Mariani et al.’s research [3]. Burke, the author of one of the meta-analyses referred to above, is also interested in studying the relationship between training effectiveness and the degree of trainee involvement. The authors show that the interaction between the safety climate and learning leads to improved safety outcomes. Again, they argue that bespoke and engaging safety training translates learning into practice and allows workers to move in the desired direction.

Ricci et al. [4] present evidence that active teaching methods, ongoing training, and the example set by management improved safety compliance among a sample of Italian mechanical workers. The explanatory model outlined in Erik Hollnagel’s article (see Figure 1) provides an overarching view of the evidence collected on the present subject.

Sarpy and Burke, and Ricci et al., consider distal and proximal factors, with Sarpy and Burke concentrating on outcomes in terms of safety *participation* and Ricci et al. addressing outcomes in terms of safety *compliance*. Meanwhile, Mariani et al.’s paper will prove a useful resource for those who want to evaluate levels of safety training engagement. This is the right approach if training is to be designed for safety tuning rather than apportioning blame.

Gilardi et al. [5] discuss the concept of a community of practice wherein the root causes of accidents are tackled (through cooperative learning). Structured comparison enables peers to improve their professional practice, strengthen their professional identity, and generate new knowledge. Gilardi et al. point out that safety training is more effective when it values people as a resource and not a problem. Engaging and participatory training should, therefore, be an integral part of safety tuning in the workplace. Furthermore, it can improve the competence of systems in general.

Those interested in delivering effective training with the intention of designing safer systems should think not only about quantity, but also quality. Training interventions that focus solely on content (the object of the training) are destined to fail if they do not shift their attention even toward the best methods (how to carry out the training). This conclusion may not seem particularly original; after all, the sixteenth-century philosopher Michel de Montaigne wrote in his book *Les Essais* that “To teach is not to fill up a vase, but to light a fire”.

What, then, should we include in our current research agenda? The answer is simple: studies that discuss the issues highlighted herein. However, as readers of Ricci et al.’s article will discover, only a small number of articles are published annually in the field. We need many more such contributions before researchers can make well-founded suggestions as to how workplace safety might be improved—and lives saved.

## Figures and Tables

**Figure 1:**
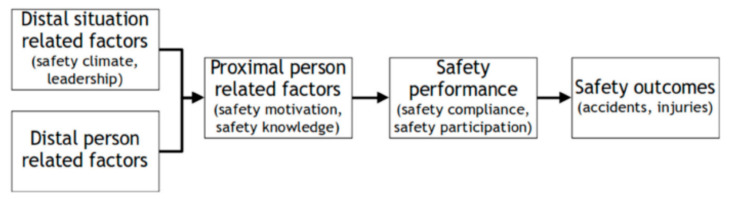
An integrative model of workplace safety (adapted by Hollnagel [1]).

## References

[1] Hollnagel E. (2021). Safer Systems: People Training or System Tuning?. Eur. J. Investig. Health Psychol. Educ..

[2] Mariani M.G., Petruzziello G., Vignoli M., Guglielmi D. (2022). Development and initial validation of the Safety Training Engagement Scale (STE-S). Eur. J. Investig. Health Psychol. Educ..

[3] Sarpy S.A., Burke M.J. (2021). An evaluation of safety training for a diverse disaster response workforce: The case of the Deepwater Horizon oil spill. Eur. J. Investig. Health Psychol. Educ..

[4] Ricci F., Panari C., Pelosi A. (2022). Safety compliance in a sample of Italian mechanical companies: The role of knowledge and safety climate. Eur. J. Investig. Health Psychol. Educ..

[5] Gilardi L., Marino M., Fubini L., Bena A., Ferro E., Santoro S., Tosco E., Pasqualini O. (2021). The Community of Practice: A Method for Cooperative Learning of Occupational Health and Safety Inspectors. Eur. J. Investig. Health Psychol. Educ..

